# Etiologies and outcome of lower gastrointestinal bleeding in patients presenting to a tertiary care Children’s Hospital

**DOI:** 10.12669/pjms.37.2.2676

**Published:** 2021

**Authors:** Muhammad Abu Talib, Muhammad Tariq Aziz, Hassan Suleman, Ghazi Khan Khosa, Saima Jabeen Joya, Ibrar Hussain

**Affiliations:** 1 Muhammad Abu Talib, Department of Pediatric Gastroenterology and Hepatology, Children’s Hospital and The Institute of Child’s Heath, Multan, Pakistan; 2 Muhammad Tariq Aziz, Department of Pediatric Gastroenterology and Hepatology, Children’s Hospital and The Institute of Child’s Heath, Multan, Pakistan; 3 Hassan Suleman, Department of Pediatric Gastroenterology and Hepatology, Children’s Hospital and The Institute of Child’s Heath, Multan, Pakistan; 4 Ghazi Khan Khosa, Department of Pediatric Gastroenterology and Hepatology, Children’s Hospital and The Institute of Child’s Heath, Multan, Pakistan; 5 Saima Jabeen Joya, Department of Pediatric Gastroenterology and Hepatology, Children’s Hospital and The Institute of Child’s Heath, Multan, Pakistan; 6 Ibrar Hussain, Department of Pediatric Gastroenterology and Hepatology, Children’s Hospital and The Institute of Child’s Heath, Multan, Pakistan

**Keywords:** Anemia, Colonoscopy, Lower Gastrointestinal Bleeding, Polyps, Colitis

## Abstract

**Objective:**

To determine etiology and outcome of children with lower gastrointestinal bleeding (LGIB).

**Methods:**

This was a prospective study conducted at the Department of Pediatric Gastroenterology and Hepatology, Children’s Hospital and The Institute of Child’s Heath, Multan, Pakistan, from July 2019 to March 2020. A total of 148 cases presented with bleeding per rectum and underwent colonoscopy, were included. Children of both genders and aged three month to 15 years were included. Detailed history, clinical examination, laboratory studies, colonoscopy and histopathology were done in all cases. Study information like demographics, complaints, general clinical examination, colonoscopy and histopathological findings were recorded.

**Results:**

Overall, mean age was noted to be 7.20±1.83 years. Abdominal pain was reported in 41 (27.7%), diarrhea 36 (24.3%), fever 12 (8.1%) and constipation in 4 (2.7%). Pallor was noted among 68 (45.9%), weight loss 39 (26.3%) and tachycardia 31 (20.9%). Colonoscopy revealed juvenile colonic / rectal polyps, infectious colitis and solitary rectal ulcer (SRU) as the most common etiologies found among 58 (39.2%), 20 (13.5%) and 19 (12.8%) cases respectively. Juvenile polyps and non-specific colitis were the commonest histopathological findings seen in 55 (37.2%) and 20 (13.5%) cases respectively. Colonoscopic polypectomy was used to remove all juvenile polyps.

**Conclusion:**

LGIB is presentation of various underlying causes. Children with LGIB commonly present with abdominal pain. Juvenile polyps were the most frequent cause of LGIB among children flowed by non-specific colitis. Most of the children having LGIB were diagnosed and treated successfully, few are in remission and very few were found resistant to treatment.

## INTRODUCTION

GI bleed is known as upper GI bleed (UGIB) and lower GI bleed (LGIB) as per site of bleeding. Lower gastrointestinal bleeding (LGIB) is described as bleeding distal to the ligament of treitz and present as rectal bleeding.^1^ Pathologies related to mucosa and vasculature of gastrointestinal (GI) tract may cause GI bleeding which is thought to be a serious presentation at any age.^2^ LGIB usually present as hematochezia, melena, occult bleeding having symptoms of tiredness and pallor or symptoms depicting severe blood loss like malaise, tachycardia or shock.^3^

Causes of LGIB differ among children from adults. Simple causes like anal fissure or juvenile polyps need little or no treatment while occasionally these may complicate some serious underlying abnormalities like intussusception, Meckel’s diverticulum and midgut volvulus.^4^,^5^ Anemia accompany children with chronic LGIB whereas identification of the source of bleeding is considered very important to form a management strategy.^6^ Among children history, detailed physical examination of perianal area, digital rectal examination and stool test may help in individuals having usual causes of per rectal bleeding. Diagnostic evaluation includes endoscopy, radiography, technetium-labeled red blood cells scans as well as angiography. After confirmation of bleeding as LGIB, proctosigmoidoscopy followed by colonoscopy is recommended among all cases for further evaluation and diagnosis.^3^,^7^

Colonoscopy is proved to be a safe and effective tool for investigating lower GI pathologies among children.^8^ Advantages of colonoscopy included direct visualization and identification of the source of bleeding, numerous management possibilities and effective diagnostic as well as therapeutic tool. Colonoscopy is needed for the confirmatory diagnosis of LGIB. On the other hand, colonoscopy need colon preparation, sedation, equipped endoscopy facility, skilled staff and invasive nature.^9^,^10^

Etiologies of LGIB vary among children of different geographies.^1^,^3^ To assist clinicians managing children with LGIB, epidemiological studies are needed. As no study has been done in South Punjab region of Pakistan to evaluate the etiology and outcome of LGIB among children, this study was planned to determine frequent etiologies and outcome of children with LGIB.

## METHODS

This prospective study was done at Department of Pediatric Gastroenterology and Hepatology, Children’s Hospital and The Institute of Child’s Heath, Multan, Pakistan, from July 2019 to March 2020. A total of 148 cases presented with bleeding per rectum and underwent colonoscopy, were included. Children of both genders and aged three months to 15 years were included. Those cases that required emergency surgery or ingested red meat, or peroxidase containing fruits/vegetables like broccoli, cantaloupes or turnips in the past 48 hours were not included.^11^,^12^ Children having bleeding disorders, infectious diseases leading to thrombocytopenia (like dengue fever, viral infections), or chronic liver disease were also excluded.

Approval from Institute’s Ethical Committee was sought (Dated: 08-07-2019, Ref. No. ERC/118) and informed consent from parents/guardians of all the study participants were taken.

Complete history taking and clinical examination as well as laboratory studies, colonoscopy and histopathology were done in all cases. Colonoscopy was performed by consultant Paediatric gastroenterologist of the same department. All cases enrolled had received 24 hours of bowel preparation before colonoscopy while standard general anesthesia was employed for colonoscopy procedure. Several mucosal biopsies were taken under direct visualization of mucosa and vasculature. Biopsy samples were kept under phosphate-buffered formalin for fixing and sent to institutional laboratory for the review.^13^ Colonoscopic polypectomy was used to remove all polyps and polyps were sent for histopathology.

SPSS version 20.0 was used for data handling and statistical analysis. All the study data including age, gender, associated complaints, general clinical examination, colonoscopy and histopathological findings were recorded on a specifically designed proforma with the help of hospital medical record files of the cases. Quantitative data like age was represented in terms of mean and standard deviation while qualitative data like gender, associated complaints, general clinical examination, colonoscopy and pathological findings were shown as frequency and percentages.

## RESULTS

Out of a total of 148 cases, 83 (56.1%) were male and 65 (43.9%) females. Overall, mean age was noted to be 7.20±1.83 years. Most of the children, 90 (60.8%) were between three to six years of age, 28 (18.9%) six to nine years and remaining 30 (20.3%) between 9 to 15 years.

In terms of commonest complaints in the present study, abdominal pain was reported in 41 (27.7%), diarrhea 36 (24.3%), fever 12 (8.1%) and constipation in 4 (2.7%). Table-I enlist most common general clinical examination findings as pallor was noted among 68 (45.9%), weight loss 39 (26.3%) and tachycardia 31 (20.9%).Table-I. There were 95 (64.2%) cases that had anemia at the time of admission.

**Table-I:** Frequency of General Clinical Examination findings among children with LGIB (n=148).

*General Clinical Examination*	*Number (%)*
Pallor	68 (45.9%)
Weight Loss	39 (26.3%)
Tachycardia	31 (20.9%)
Hypotension	26 (17.6%)
Normal	45 (30.4%)

Details of Colonoscopic findings are shown in Table-II. Colonoscopy revealed juvenile colonic / rectal polyps, infectious colitis and solitary rectal ulcer (SRU) as the most common etiologies found among 58 (39.2%), 20 (13.5%) and 19 (12.8%) cases respectively. Cow’s milk protein allergy (CMPA) was found among 11 (7.4%) while 2 (1.4%) children were noted to have meckel’s diverticulum.

**Table-II:** Colonoscopic findings among children having LGIB (n= 148).

*Colonoscopy Findings*	*Number (%)*
Juvenile colonic / rectal polyps	58 (39.2%)
Infectious Colitis	20 (13.5%)
Solitary Rectal Ulcer (SRU)	19 (12.8%)
Inflammatory Bowel Disease	15 (10.1%)
Suspected Abdominal Tuberculosis	7 (4.7%)
Eosinophilic Colitis	3 (2.0%)
Idiopathic	26 (9.0%)

Frequency of various histopathological findings are shown in Table-III. Juvenile polyps were the commonest histopathological finding noted among 55 (37.2%) children.

**Table-III:** Frequency of histopathological findings among children with LGIB.

*Pathological Findings*	*Number (%)*
Juvenile Polyps	55 (37.2%)
Familial adenomatous polyposis	3 (2.0%)
Crohn’s Disease	3 (2.0%)
Solitary Rectal Ulcer (SRU)	19 (12.8%)
Ulcerative Colitis	12 (21.6%)
Post Infection Colitis	20 (13.5%)
Non specific colitis (idiopathic)	20 (13.5%)
Eosinophilic Colitis	3 (2.0%)
Caseating granulomatous inflammation (Abdominal TB)	7 (4.7%)
Normal Findings (idiopathic)	6 (4.1%)

Among 58 polyps cases, 55 were having juvenile polyps (non-malignant) and three were familial adenomatous polyposis (FAP). All juvenile polyps were found to be in rectosigmoid region and solitary. Colonoscopic polypectomy was used to remove all juvenile polyps. Among 55 juvenile polyps cases, 53 became disease free while bleeding recurrence reported in two cases. All three FAP cases were referred for surgery and colectomy was done (one death reported while two are on follow up). All infectious colitis children were treated successfully with antibiotics for 10 days while no recurrence was found during the follow up. All 19 cases of SRU were treated with hydrocortisone enema, sitz bath and diet modification, seven cases were partially responsive, 10 fully responsive (no recurrence noted during follow ups) while two cases were noted to be resistant to treatment. In 15 cases of IBD, 12 had ulcerative colitis who were treated with steroid, aminosalicylic acids, probiotics and enemas while three cases had crohn’s disease (all are on follow up with partial remission). Seven children were found to have ileocolonic tuberculosis (TB) as histopathological examination revealed caseating granulomatous inflammation as well as positive staining for AFB on Ziehl-Neelsen stain. All abdominal TB cases were responsive to treatment and are on follow up. All three cases of Eosinophilic colitis were treated with steroid, montelukast, hypo allergic diet while no recurrence found. All patients with idiopathic findings were treated with antibiotics, hypo allergic diet, high fiber diet successfully except 4 who had occasional bleeding per rectum and are being investigated further). Constipation was treated with lactulose.

## DISCUSSION

LGIB is a frequent cause of referrals to Paediatric Gastroenterology Centers.^14^ It was noted in the current study that male children predominated the scenario and of included patients, 56.1% were male. Deeb MM et al. from Egypt^15^ as well where 68.0% of the cases with LGIB were male. A study from India,^16^ showed that male to female ratio was 2.16:1 among children undergoing colonoscopy with LGIB. Mean age of patients in our study was 7.20±1.83 years while most. It has been noted previously as well that children of younger age more frequently present with LGIB.^15^,^16^ A study from Iran^13^ revealed that 79.9% of the children presenting with LGIB were less than 10 years of age. A study done by Mandhan P et al.^17^ also noted younger age to be the commonest among children with LGIB.

In present study, abdominal pain was the commonest presenting complaint reported in 27.7%, diarrhea 36 (24.3%), fever 12 (8.1%) and constipation in 4 (2.7%). Findings of our study were very similar to a study in Egypt^15^ with abdominal pain and diarrhea being commonest presentations. Arvola T et al.^18^ noted anemia, abdominal pain and diarrhea to be the commonest presentations in children with rectal bleeding. Ojuawo A and Colleagues^19^ noted diarrhea, vomiting and abdominal pain to be the commonest among patients having LGIB. Zahmatkeshan M et al from Iran^13^ reported fever, abdominal pain and diarrhea to be commonest symptoms accompanying bloody stool. We also noted that 45.9% of our cases had pallor when general clinical examination was done. Similar findings have been seen by other researchers as well.^15^

We found 64.2% of the cases to have anemia at the time of admission. These findings correlate to another study where 61% of the cases with LGIB had anemia.^15^ Anemia has been noted to be a common finding among children having chronic blood loss.^20^

In this study, colonoscopy revealed juvenile colonic / rectal polyps to be the commonest finding, noted among 39.2% cases. In a study from Egypt,^15^ similar findings were observed with polyp being commonest Colonoscopic finding, in 44% whereas results from a study done by Clarke G et al.^14^ revealed that polyps were only seen in 10% of the cases. Higher frequencies of polyps (75%) on colonoscopy among children having LGIB was shown by Mandhan P^17^ Studies done by Balkan E et al.^21^ noted 53% having rectal polyps while studies from many parts of the world identified rectal polyps to be the commonest etiology among children with LGIB.^8^,^22^,^23^

In the present analysis, juvenile polyps turned out to be the commonest pathological finding, noted among 37.2%, non-specific colitis in 13.5% and ulcerative colitis in 21.6%. Deeb MM et al.^15^ from Egypt also noted juvenile polyps to be the most frequent pathological finding. These polyps are normally hamartomatous and responsible for about 90% of all kind of polyps noted among children.^6^ A local study done by Wajeehuddin AR^12^ also found rectal polyps to be the commonest pathological finding which is very similar to what was found in the present study. Ulcerative colitis is another very common etiology of rectal bleeding as was found in our study. Previous publications reveal that around 20-25% of the children with LGIB had ulcerative colitis.^10^,^24^ It is also a well-known phenomenon that most of the cases with ulcerative colitis present with rectal bleeding. GI Infections are also known to be a common cause of LGIB among children as was seen in this study. Non-specific colitis was seen in 20% of the cases in a study done earlier.^15^ Lesions are usually confined to rectum but may extend proximally to engage sigmoid colon.^25^

We noted that 4.1% of the cases had normal pathological findings. Previous findings also revealed that about 6-10% of the children having LGIB represent normal pathological findings.^13^,^15^

### Limitation of the study

Most of the children in our setting were referred cases so this could mean that we evaluated cases which had serious conditions that could influence the bias in terms of selection of the cases. Yet, present study is among the very few studies evaluating children with LGIB. As this was a single center study, it is suggested that more studies involving different set of populations and multiple centers needs to be conducted to further add to whatever is known regarding children presenting with LGIB in Pakistan.

## CONCLUSION

LGIB is presentation of various underlying causes. Children with LGIB commonly present with abdominal pain. Juvenile polyps was the most frequent cause of LGIB among children flowed by non-specific colitis. Colonoscopy is a useful and safe procedure in children for evaluation of LGIB both for diagnostic and therapeutic purposes. Most of the children having LGIB were diagnosed and treated successfully, few are in remission and very few were found resistant to treatment.

### Author’s Contribution:

**MAT:** Data Collection, Drafting and accountable for the accuracy or integrity of the work.

**MTA:** Supervision, Proof Reading.

**HS:** Study Design, Data Interpretation.

**GKK:** Data Analysis, Discussion.

**SJJ, IH:** Literature Review, Introduction.
